# The Nature of Abstract Orthographic Codes: Evidence from Masked Priming and Magnetoencephalography

**DOI:** 10.1371/journal.pone.0010793

**Published:** 2010-05-25

**Authors:** Liina Pylkkänen, Kana Okano

**Affiliations:** 1 Department of Linguistics, New York University, New York, New York, United States of America; 2 Department of Psychology, New York University, New York, New York, United States of America; 3 Department of Psychology, Tufts University, Medford, Massachusetts, United States of America; University of Leuven, Belgium

## Abstract

What kind of mental objects are letters? Research on letter perception has mainly focussed on the visual properties of letters, showing that orthographic representations are abstract and size/shape invariant. But given that letters are, by definition, mappings between symbols and sounds, what is the role of sound in orthographic representation? We present two experiments suggesting that letters are fundamentally sound-based representations. To examine the role of sound in orthographic representation, we took advantage of the multiple scripts of Japanese. We show two types of evidence that if a Japanese word is presented in a script it never appears in, this presentation immediately activates the (“actual”) visual word form of that lexical item. First, equal amounts of masked repetition priming are observed for full repetition and when the prime appears in an atypical script. Second, visual word form frequency affects neuromagnetic measures already at 100–130 ms whether the word is presented in its conventional script or in a script it never otherwise appears in. This suggests that Japanese orthographic codes are not only shape-invariant, but also script invariant. The finding that two characters belonging to different writing systems can activate the same form representation suggests that sound identity is what determines orthographic identity: as long as two symbols express the same sound, our minds represent them as part of the same character/letter.

## Introduction

Letters are the entry point to linguistic processing in reading. Thus understanding the nature of orthographic representations is fundamental to models of visual language comprehension. Two decades of research on letter perception have established that orthographic representations are abstract [1], [2]. One of the most useful methods for demonstrating this has been the masked priming paradigm, which assesses the effect of subliminally presented primes on target processing [3]. In this paradigm, semantic effects are not generally obtained, suggesting that masked priming reflects earlier processing stages, specifically, the activation of orthography and the visual form of the word [4], [5], [6]. In masked priming, equal amounts of identity priming are obtained whether or not the prime matches the target in case and whether or not the cross-case representations are visually similar (kiss/KISS vs. edge/EDGE) [7]. Thus letters are a classic case of shape invariant visual representations.

What is the definition of “a letter” then, as a mental representation? The masked priming literature suggests that a letter is the abstract entity that unites all possible visual forms of the letter, independent of size and font [8, although see also 9]. But what is less well understood is the role of sound in this account. If an upper case ‘K’ and a lower case ‘k’ are part of the same letter because they both map onto the same sound/k/, then ‘c’ should be part of that letter, too, since ‘c’ and ‘k’ can both stand for/k/(e.g., cat vs. kit). But this is not the native intuition–any English speaker would say that ‘k’ and ‘c’ are different letters.

Whether abstract letter representations are defined on the basis of sound is difficult to test in English, because of the rather complicated relation between sound and orthography. However, languages with multiple scripts provide a unique opportunity to assess the role of sound in abstract orthographic representation. For example, in Japanese, which employs two different syllabaries, each syllable can be expressed either by a katakana character or by a hiragana character. Do the katakana and hiragana versions of a single syllable activate/share the same representation or do they map onto two distinct character representations?

We addressed this question with a combination of behavioural and neuromagnetic measures. In Japanese, although each syllable can be expressed in either katakana or hiragana, many words are conventionally only spelled in one syllabary. Specifically, hiragana is generally used for Japanese words that cannot be written in kanji (Chinese characters used in Japanese writing) and to inflect kanji, whereas katakana is used to spell out loan words. Given the different roles of the two scripts, we can ask whether, for example, a hiragana version of a katakana word would activate the abstract form representation of that word just as quickly as presenting the word in its typical script. If sameness of sound is the crucial factor, then the hiragana stimulus should immediately and directly activate the abstract form representation of the word. In contrast, if abstract letter identities are purely orthographic, non-sound-based, and script specific, then presenting a word in an atypical script should be a poor activator of a word's abstract form representation.

As one test of these predictions, we conducted a behavioural masked priming study, assessing whether equivalent repetition priming would be obtained for within script and across script prime-target pairs. Crucially, we needed to eliminate any possibility of semantic priming, and thus primes were presented quickly enough to block an effect of semantic relatedness, which was independently assessed with a semantic control condition. As targets, we used katakana words that had zero written frequency in hiragana according to Amano & Kondo [10]. In the script change condition, the prime was the hiragana version of the katakana target, and in the full repetition condition, the prime was identical to the target. If the hiragana and katakana primes are equally good activators of the abstract form representation of the target, then an equivalent priming effect should be obtained in both cases.

As just mentioned, in our masked priming study, we presented the primes briefly enough to disallow semantic priming. Thus priming effects for the full repetition and script change conditions can be interpreted as reflecting priming at a pre-semantic level. In a second experiment, we conducted a more direct test of the processing level at which form/character representations may be script independent. In order to track visual word form processing stage by stage, we took advantage of the millisecond temporal resolution of magnetoencephalography (MEG). In response to visual words, MEG shows two prominent early responses, the M100 and the M170. The M100 (also called M130 or “Type 1 activity”) was initially shown to be sensitive only to low level stimulus properties such as size and luminance [11], [12], [13], but recent studies have also shown M100 sensitivity to orthographic properties such as letter string frequency and transition probability from one string to the next [14; see also 15 for converging evidence from EEG]. Thus the M100 is the first potential correlate of activity sensitive to letters and letter strings. The subsequent M170 stage (N170 in EEG), on the other hand, is thought to reflect access to stored visual word forms [16], or, as suggested by recent MEG studies on morphologically complex words, access to stored representations of morpheme forms [14], [17], [18]. Crucially, neither the M100 nor the M170 show any true lexical effects, i.e., there is no evidence that contact with the semantic lexicon has been made at these stages [18].

Our aim was to assess whether either the M100 or the M170 would show evidence of script-invariant, abstract character representations. An MEG version of our masked priming experiment might be one way to achieve this; however, this method is complicated by the fact that in a masked priming design, the M100s and M170s elicited by the prime and the target necessarily overlap, making the data difficult to interpret. It is known that in English, MEG effects of masked repetition priming are obtained at around ∼225 ms after target onset [19]. However, this effect is difficult to interpret functionally or relate to known visually evoked MEG components, due to the blurring of responses just mentioned.

To avoid the complications of using masked priming in MEG, we instead manipulated the lexical frequency of typical katakana words and presented them in both katakana and in hiragana in a single word lexical decision task. Since lexical frequency correlates heavily with both the visual form frequency of a word as well as lower level frequency, such as bi- and trigram frequency, frequency should have a relatively early effect for the typically presented katakana stimuli, possibly already at the M100. The question though is, when should frequency affect the processing of the atypically presented hiragana stimuli? If the form representation activated by these stimuli is the same as the one activated by the typically presented katakana stimuli, then the effect of frequency should emerge equally early for the two presentations. However, if the abstract form representations of these katakana words are non-sound-based and katakana-specific, then the hiragana versions should not activate these representations. On this account, an effect of frequency should not be observed at the level of form representations for the hiragana presentations, but rather sometime later, at the level of phonological or semantic access. In sum, in our second experiment, we took advantage of the millisecond temporal resolution of magnetoencephalography (MEG) to assess whether typically and atypically presented words would elicit an effect of frequency equally early. If orthographic symbols are sound based, then regions associated with the activation of abstract visual word forms should show the same effect of frequency in both cases.

### Detailed hypotheses and predictions

#### Experiment 1a: Masked priming

If abstract letter representations are sound-based, the form representation of a word should be activated equally quickly whether or not a word appears in its typical script/orthography. Experiment 1a assessed this in a lexical decision task using Japanese katakana words which were either preceded by the same word in katakana or by an atypical hiragana version of the word. Primes were masked in order to diagnose subliminal activation of pre-semantic form representations. To maximally prevent semantic priming, we used a short stimulus onset asynchrony of 39 ms, which in previous studies has not produced semantic priming [e.g., 5]. To further rule out a semantic account of our findings, our design included a semantic control condition.

#### Experiment 1b: Unmasked priming

Experiment 1b was a control experiment for Experiment 1a, aimed at testing whether the semantic primes used in Experiment 1a would yield a reliable priming effect with a longer SOA (300 ms).

#### Experiment 2: Magnetoencephalography

In our MEG experiment, we moved away from the traditional masked priming paradigm (for the reasons discussed above) and instead manipulated the written frequency of katakana word forms. Again, the critical stimuli were words that typically appear in katakana and have zero written frequency in hiragana. These stimuli were divided into high and low frequency bins, and then presented to the subjects in both katakana and hiragana. We predicted that if the form representation activated by the katakana and the unfamiliar hiragana primes is truly the same, then the frequency of that shared form should have an equally early effect on the processing of both scripts. In contrast, if the form representations of these words are non-sound-based and katakana specific, then the hiragana presentations should elicit an effect of frequency later than the katakana presentations, at the level of phonological/semantic access.

One previous EEG study has already investigated the processing profile of atypically presented Japanese words [20]. This study used words that are typically expressed in kanji, a logographic script, and tested whether visually unfamiliar katakana versions of these words would elicit the same, well-documented, left-lateral N170 that is generally observed for visual word form presentation. The unfamiliar katakana forms showed a similarly left-lateral N170 as familiar word forms. The authors concluded that the N170 is driven by character familiarity, not by familiarity to particular word forms. However, the sound-based hypothesis examined here offers another explanation of this finding. Given that the unfamiliar katakana forms have the same sound representation as the familiar kanji forms, the sound-based hypothesis would predict these forms to activate the same abstract, script-invariant, orthographic code.

As described above, our study differs from [20] in that in addition to script typicality, we varied form frequency (or familiarity), with the goal of assessing whether the atypically presented stimuli would nevertheless elicit an early effect of form familiarity, suggesting that they directly activate the abstract form representation of the word. The time-course of frequency effects was assessed with MEG. As summarized earlier in this introduction, the MEG equivalent of the N170 ERP is the visual M170, generated in ventral regions of posterior temporal cortex including the Visual Word Form Area [21], [22]. Consistent with the ERP literature, the M170 has been proposed as the relevant MEG response for abstract encoding of visual word form representations [18], [17]. Thus form frequency might affect M170 activity. Interestingly, several imaging studies have reported that the generating region of the M170, i.e., the Visual Word Form Area, shows activation even for auditory word presentation [23]. This finding conforms to the sound-based hypothesis pursued here since on the sound-based account, visual word forms are defined on the basis of sound representations. Thus one might expect sound representations to automatically activate the corresponding abstract orthographic forms.

Another possible locus for an early frequency effect is the visual M100. An effect at the M100 would most likely reflect an effect below the word form level, perhaps at the level of bigrams or trigrams, given the sensitivity of the M100 to orthographic frequency and transition probability [14]. Although our corpus [10] allowed us to control for character frequency, our high and low frequency bins were likely to differ in bi- and trigram frequency, which heavily correlate with lexical frequency. Prior EEG studies have also reported effects of lexical frequency at around ∼120 ms [15], [24], [25], further enforcing the possibility of us detecting a frequency effect already in the M100 time window.

In sum, we analyzed both the M100 and the M170 to assess which of these components would show effects of word form frequency for our typically presented stimuli and whether the atypical stimuli would show the same or a later effect.

## Methods

### Experiment 1a: Masked priming

#### Participants

Eight native Japanese speakers, residing in New York City at the time of the experiment, participated in the study (7 female). The participants' ages ranged from 24 to 46 (mean age 32.5). All participants had normal or corrected to normal vision and were graduates of high school level or above with Japanese as the sole language of instruction. None of the participants reported language or reading disorders. All procedures were approved by New York University's Committee on Activities Involving Human Subjects and informed written consent was obtained from each participant.

#### Materials

The critical materials consisted of 45 sets of four prime-target pairs, with the conditions repetition, script change, semantic, and unrelated. The target stayed constant within a set, while the prime varied. The target was always a two to five character katakana word, i.e., a word that is typically written in the katakana syllabary. All targets had zero frequency in both hiragana and kanji, according to [10]. In the repetition condition, the prime was identical to the target. In the script change condition, the prime was the target word, except now written in the hiragana syllabary. The semantic condition involved a prime that was semantically related to the target, but phonologically, orthographically, and morphologically unrelated. The unrelated primes were phonologically, orthographically, morphologically, and semantically unrelated to the targets. Table 1 depicts examples of each stimulus type and Appendix S1 lists all the critical materials. The test materials were combined with 180 prime-target pairs where the target was a nonword and the prime was a real word in an atypical script. The nonwords were created by switching around the letters in the critical words to match for character frequency across words and nonwords. Each prime was preceded by a forward mask consisting of a row of five Xs (XXXXX). Then the prime appeared for 39 ms, and was immediately followed by the target. The design was within-subjects and stimulus presentation was constrained in such a way as to counterbalance the effect of target repetition across conditions. Other than this constraint, stimulus order was random.

**Table 1:** Example stimuli for Experiments 1a and 1b.

Condition	Prime	Target
Repetition	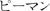 piman (pepper)	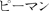 piman (pepper)
Script change	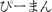 piman (pepper)	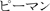 piman (pepper)
Semantic	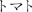 tomato (tomato)	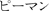 piman (pepper)
Unrelated	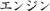 ennjin (engine)	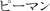 piman (pepper)

#### Procedure

Presentation of the stimuli and the recording of lexical decision times were controlled by PsyScope X, utilizing an external button box. Each trial began with a forward mask of five ex-marks (XXXXX), presented for 500 ms. Next, the prime appeared for 39 ms. Target presentation followed immediately. The target stimulus stayed on the screen until the subject's button press response. The intertrial interval was 500 ms. Participants performed lexical decisions to the targets with their left hand, using the index finger for words and the middle finger for nonwords. The participants were instructed to respond as quickly and as accurately as possible. The participants were not informed of the presence of the prime. Each participant received a total of 12 practice trials prior to the actual experiment.

### Experiment 1b: Unmasked priming

#### Participants

Eight native Japanese speakers, residing in New York City at the time of the experiment, participated in the study (7 female). None of the participants had participated in Experiment 1a.

The participants' ages ranged from 21 to 34 (mean age 28.5). All participants had normal to corrected to normal vision and were graduates of high school level or above with Japanese as the sole language of instruction. None of the participants reported language or reading disorders. All procedures were approved by New York University's Committee on Activities Involving Human Subjects and informed written consent was obtained from each participant.

#### Materials

The materials were identical to those of Experiment 1a.

#### Procedure

The procedure was identical to that of Experiment 1a, except that prime duration was 300 ms. Thus the primes were clearly visible to the subjects.

### Experiment 2: Magnetoencephalography

#### Participants

Sixteen native Japanese speakers (14 female) currently residing in New York City participated in the study. Participants' ages ranged from 21 to 42 (mean age 33.8). All participants were right handed, had normal or corrected to normal vision, and were graduates of high school level or above with Japanese as the sole language of instruction. None of the participants reported language or reading disorders. All procedures were approved by New York University's Committee on Activities Involving Human Subjects and informed written consent was obtained from each participant.

#### Materials

Sixty Katakana words served as the target stimuli; thirty were low frequency (range 2–99, mean 39.5) and thirty high frequency (range 436–8842, mean 1860.1), according to the *Lexical properties of Japanese* [10]. All words were typically written in Katakana, and had zero frequency in both Hiragana and Kanji [10]. A 2×2 design was employed, crossing frequency (Low vs. High) with presentation script (Typical vs. Atypical). In the typical conditions, the Katakana words were presented in Katakana, i.e., in their typical script. In the atypical conditions, the same Katakana words were written in the Hiragana syllabary, which resulted in visually unfamiliar forms. The 2×2 design is illustrated in Table 2 with examples. Appendix S2 includes a full list of the stimuli.

**Table 2:** Design of Experiment 2.

	Katakana (typical)	Hiragana (atypical)
Low Frequency	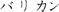 barikan (electric shaver)	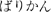 10barikan (electric shaver)
High Frequency	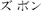 zubon (trousers)	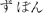 zubon (trousers)

A summary of the lexical statistics of the stimuli is presented in Table 3. The high and low frequency words were matched for length and character frequency [10]. Imageability ratings were not available for the Japanese words but we assessed imageability by translating the words to English and then using the MRC Psycholinguistic Database, according to which the English translations did not differ in imageability. We aimed to used maximally unambiguous words; none of the target stimuli were homonyms or obvious polysemes. Further, given that our subjects were residing in an English speaking environment, we made sure that none of the target words were of English origin, which might have induced lexical access of the corresponding English word. All words were monomorphemic. Stimuli were presented in a pseudorandom order such that the effect of across-script repetition was counterbalanced across the low and high frequency conditions.

**Table 3:** Experiment 2 stimulus properties.

	Low	High	T-test
Frequency (SD)	39.5 (29.9)	1860.1 (2169.7)	*t*(58) = −4.6, *p*<0.001
Character frequency (SD)	985.2 (573.2)	1095.9 (749.6)	*t*(58) = −0.64, *p* = 0.52
Length (SD)	3.7 (0.8)	3.7 (0.9)	*t*(58) = 0.15, *p* = 0.88
Imageability (SD)	547.1 (64.6)	540.4 (77.3)	*t*(58) = 0.36, *p* = 0.71

The target stimuli were embedded in a larger lexical decision experiment, which altogether involved 360 words, half of which occurred in the Katakana syllabary and the other half in Hiragana, and 180 nonword items, half of which employed Katakana and the other half Hiragana. All nonwords were pronounceable.

#### Procedure

During the experiment, subjects lay in a dimly-lit magnetically-shielded room. The stimuli were projected onto a screen at a distance of 17 cm from the subject, in MS P Mincho font (font size = 90) against a black background. Trials began with a fixation cross in the center of the screen, presented for 500 ms. This was followed by a blank screen for 300 ms, after which a word/nonword appeared, prompting a lexical decision. The stimulus stayed on the screen until the subject's button press response. Participants indicated word decisions with their left index finger and nonword decisions with their left middle finger. Each participant received twelve practice trials prior to the relevant experiment.

Neuromagnetic fields were collected using a 275-channel whole head axial gradiometer (CTF, Vancouver, Canada) sampling at 600 Hz in a band between 0.1 Hz and 200 Hz. The experiment lasted about 30 min.

#### MEG data analysis

MEG data were first cleaned of artifacts and trials on which the subject responded incorrectly. The data were then averaged by condition using an epoch length of 600 ms, including a pre-stimulus interval of 100 ms. On average 7.13% of each subject's data were excluded due to errors or artifacts (SD 7.79%). Before source modelling, MEG averages were high-pass filtered at 1 Hz and low-pass filtered at 40 Hz.

As stated above, the analysis focussed on the visual M100 and M170 components, which constitute the earliest clearly detectable peaks in the evoked MEG response to visual words. The M100 is generated in midline occipital regions and peaks around 100–140 ms. The M170 is generated bilaterally in posterior occipito-temporal cortex and peaks at 170–200 ms.

The current generators of M100 and M170 activity were modelled for each subject as discrete dipole sources in BESA 5.1, using that subject's grandaverage response to all the 360 word stimuli presented in the course of the experiment. This provided a maximal signal-to-noise ratio for the source modelling and ensured that the solutions were not biased towards any particular condition. These source solutions were then fit to the individual conditions, to assess variation in peak latency and amplitude.

The dipole models were multiple source models created at the peaks of the M100 and M170 components, using data from all the sensors. In many cases, the source solutions included dipoles other than the M100 and M170 sources, but these dipoles, reflecting co-active sources, did not exhibit any particular consistency of location across subjects and were not analyzed. Twelve of the sixteen subjects exhibited a canonical M100 field pattern, and were included in the M100 analysis. M170 fields were observed for fourteen subjects; seven of these localized bilaterally, 6 left-laterally, and only one right-laterally. The goodness of fit of the M100–M170 solutions averaged at 72% for the 100–250 ms time window, with no differences between conditions (F(3, 15)<1). Notice that this goodness of fit was assessed for the same time window for all subjects, without adjustment to individual component latencies. At the M100 and M170 peaks, goodness of fits were generally at 90–95%.

Dipole peak amplitudes and latencies were assessed using the Waveforms automatic peak finder, made available by BESA (http://www.besa.de/updates/tools/matlab-scripts.php).

## Results

### Experiment 1a: Masked priming

**Figure 1:**
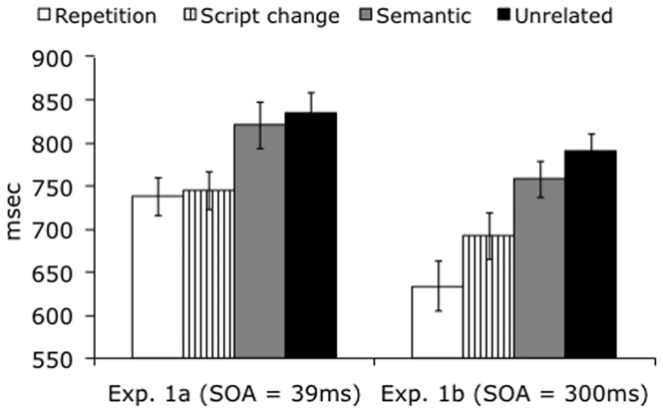
Priming data for Experiments 1a and 1b. With the short SOA (masked priming), full repetition and script change elicit an equal size priming effect, while no semantic priming is obtained. With the longer SOA of 300 ms, semantic priming is also observed.

None of the subjects reported awareness of the prime. Lexical decision data were analyzed for speed and accuracy, both by participants and by items. Mean lexical decision times per condition are depicted in Fig. 1. The by-participants analysis of reaction time data revealed a highly reliable main effect of condition (F_1_(3,7)  = 31.8, *p*<0.0001). Pairwise comparisons (Scheffe *t* test) revealed that this effect was driven by reduced reaction times to the repetition and script change conditions, in comparison to the semantic and unrelated conditions (p<0.001 for script vs. unrel, rep vs. unrel, script vs. sem, rep vs. sem). The repetition and script change conditions did not differ from each other (p = 0.9), neither did the semantic and unrelated conditions (p = 0.68). These results were replicated in the by-items analysis, which also showed a main effect of condition (F_2_(3,44)  = 37.93, *p*<0.0001), driven by faster reaction times to the repetition and script change conditions as compared to the semantic and unrelated conditions (p<0.001 for script vs. unrel, rep vs. unrel, script vs. sem, rep vs. sem; Scheffe *t* test). Again, the repetition and script change conditions did not differ from each other (p = 0.52), neither did the semantic and unrelated conditions (p = 0.34).

The accuracy data paralleled the reaction time data in that the responses to the repetition and script change conditions were more accurate (88%, SE = 4.4%, and 88%, SE = 1.9%, respectively) than the responses to the semantic (83%, SE = 3.3%) or unrelated conditions (83%, SE = 2.9%). This difference was reflected as a reliable main effect of condition in the by-items analysis (F_2_(3,44)  = 2.88, p<0.05), but not in the by-participants analysis (F(3,7)  = 2.04, p = 0.14). None of the pair-wise comparisons of the items analysis showed reliable differences.

In sum, at this very short SOA, no semantic priming was observed but the repetition and script change conditions elicited nearly identical and highly reliable priming effects. This suggests that although the atypically spelled primes of the script change condition were visually unfamiliar, they nevertheless immediately activated the abstract, pre-semantic orthographic code of the target item.

The above explanation crucially assumes that the priming in the script change condition could not be explained as a semantic priming effect. This was evidenced by the lack of semantic priming, but it still remains to be verified that these particular semantic stimuli elicit a reliable priming effect when the SOA is longer. Experiment 1b assessed this.

### Experiment 1b: Unmasked priming

Lexical decision data were analyzed for speed and accuracy, as in Experiment 1a. Reaction times showed a reliable main effect of condition in the by-participants analysis (F_1_(3,7)  = 96.97, p<0.001). The repetition and script change conditions both produced robust priming effects relative to the unrelated controls (both p's<0.001, Scheffe *t* test) as well as to the semantic condition (p<0.001). Unlike in Experiment 1a, semantic priming was now also obtained: responses in the semantic condition were on average 32 ms faster than in the unrelated condition (p<0.05). Further, although in Experiment 1a, the script change and repetition conditions elicited nearly identical reaction times, responses to the repeated stimuli were now reliably faster than responses to the script change stimuli (p<0.001), suggesting that when the subjects consciously perceived the script change, this slowed them down. This pattern of effects was replicated in the by-items analysis. The main effect of condition was reliable (F_2_(3,44)  = 83.88, p<0.001) and the related conditions were all faster than the unrelated condition in the pair-wise comparisons (p's for rep vs. unrel and script vs. unrel<0.001; for sem vs. unrel, p<0.05; Scheffe *t* test). Again, the repetition and script change conditions were also faster than the semantic condition (p<0.001), and finally, the repetition condition was faster than the script change condition (p<0.001).

Accuracy averaged at 92.8% (SE = 1.8%) for the repetition condition, at 93.6% (SE = 2%) for the script change condition, at 86.7% (SE = 1.6%) for the semantic condition and at 87.2% (SE = 2.1%) for the unrelated condition. The main effect of condition on accuracy was reliable both in the by-participants (F_1_(3,7)  = 4.24, p<0.05) and in the by-items analysis (F_2_(3,44)  = 6.4, p<0.001). No pair-wise comparisons reached significance in the by-participants analysis, but the item analysis showed a reliable difference between the semantic and repetition conditions, between the semantic and script change conditions and between the unrelated and script change conditions (all p's<0.05; Scheffe *t* test). The difference between the unrelated and repetition conditions was also marginally reliable (p = 0.06).

The results of this control experiment successfully demonstrated that the semantic materials of Experiment 1a produce reliable priming when SOA is longer.

### Experiment 2: Magnetoencephalography

#### Lexical decision data

Two participants' lexical decision time data were lost due to a technical failure. Reaction times were unusually slow, averaging over one second in all conditions (Typical High: 1233 ms; Typical Low: 1292 ms; Atypical High: 1296 ms; Atypical Low: 1402 ms). This was likely due to the changing script. Reaction times did, however, show reliable main effects of both Frequency (*F*(1, 13)  = 6.13, *p*<0.05.) and Typicality (F(1, 13)  = 23.12, p<0.001), high frequency words eliciting faster decisions and atypically presented words slower. Lexical decision accuracy averaged at 92% and 79% for the high and low frequency typically presented words, respectively, and at 87% and 71% for the atypically presented high and low frequency words. The main effects of Frequency and Typicality were both reliable with no interaction (Frequency: F(1, 13)  = 26.174, p<0.001; Typicality: F(1, 13)  = 13.63, p<0.01). Again, the rather low accuracy is likely due to the fact that subjects were asked to perform lexical decisions on stimuli that alternated in script.

#### M100 and M170 data

**Figure 2:**
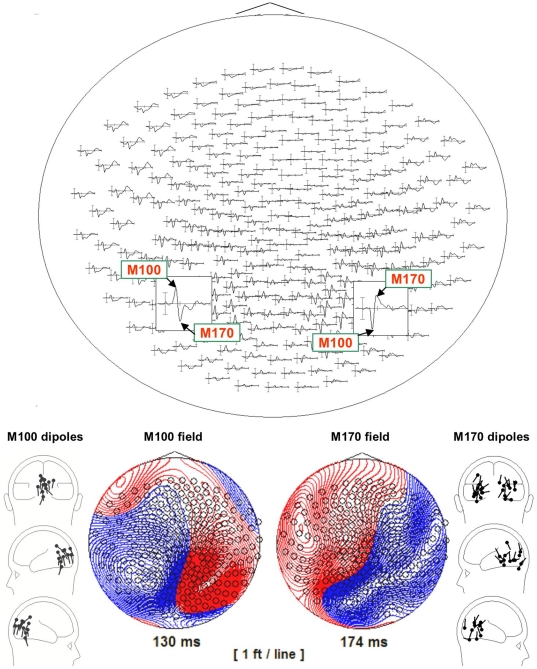
Across subjects grandaveraged response to all the critical items of Exp. 2 depicting the M100 and M170 components. The gridmap on top shows the components in sensor-space. The bottom panel depicts the field maps associated with the components. On the far left and right, the dipole localizations of all the M100s and M170s of this study are plotted within a representative subject's head frame.

Figure 2. depicts the across subjects grandaveraged response to all the critical items of this study, including the field maps of the M100 and M170 components as well as all the M100 and M170 dipole localizations for our participants. The peak latencies and amplitudes of the M100 and M170 dipoles were analyzed with 2×2 within-subjects ANOVAs with Frequency (high vs. low) and Typicality (typical katakana vs. atypical hiragana) as factors. Left and right M170 generators were analyzed separately. Mean latencies and amplitudes for these two measures are summarized in Table 4.

**Table 4:** M100 and M170 latencies and amplitudes (Experiment 2).

	Typical High	Typical Low	Atypical High	Atypical Low
M100 amplitude (nAm)	26.2	29.1	26	29.6
M100 latency (ms)	126	128	127	127
LH M170 amplitude (nAm)	17.1	19.8	19.2	20.3
LH M170 latency (ms)	181	189	190	197
RH M170 amplitude (nAm)	24.7	26	24.4	24
RH M170 latency (ms)	190	184	185	195

**Figure 3:**
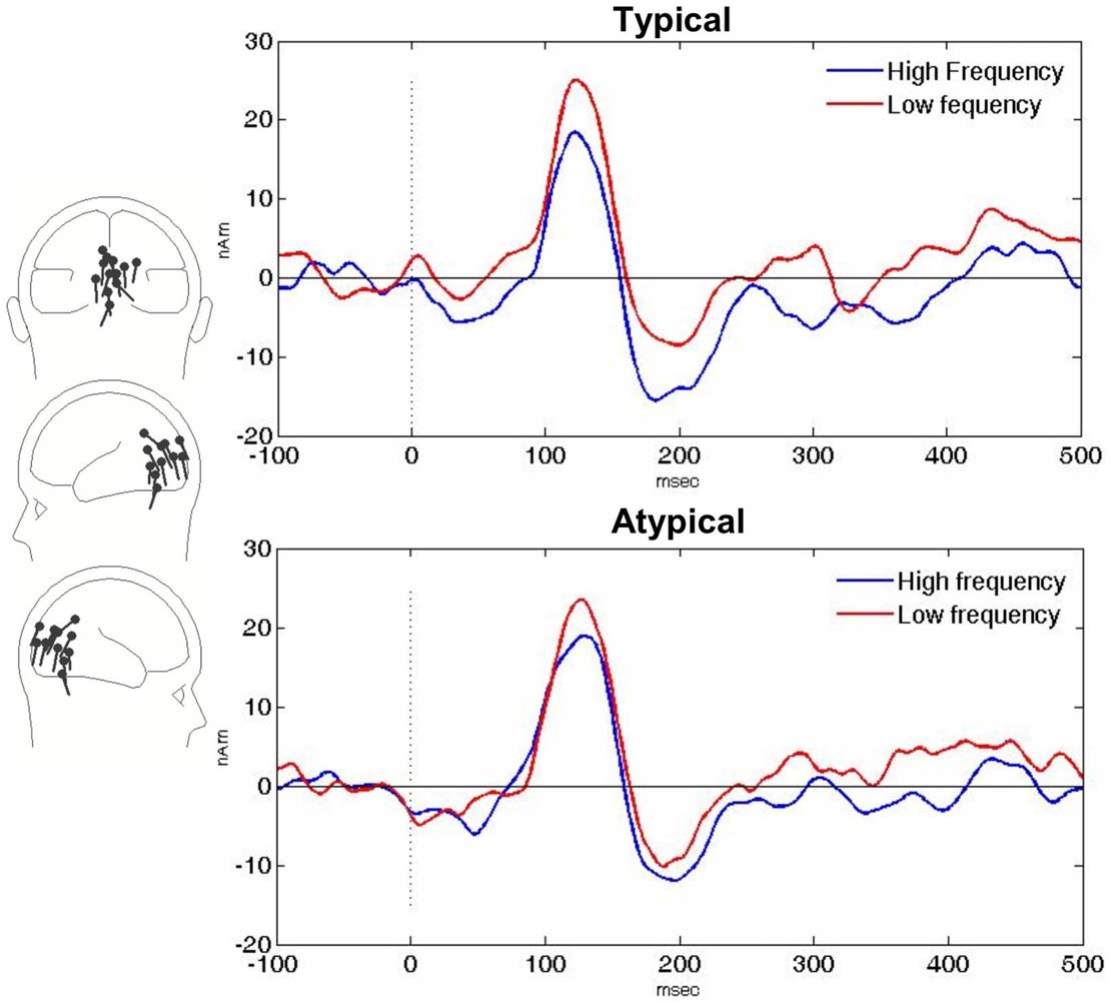
Grandaveraged M100 source waveforms illustrating a visual M100 effect of frequency both for the typically and for the atypically presented stimuli.

M100 peak latency was not affected by the manipulation. However, as shown in Figure 3, M100 amplitude was: Frequency had a reliable main effect (F(1,11)  = 5.38, p<0.05) which was not qualified by an interaction with Typicality (F(1,11)  = 0.05, p = .83). Thus we observed a similar effect of frequency both for the typically and atypically spelled items in the earliest visually evoked MEG component.

In the left hemisphere, Typicality had a reliable main effect on M170 latency, atypically presented items eliciting later peaks than typically presented items (F(1,12)  = 5.92, p<0.03). The left M170 also showed faster peak latencies for high than for low frequency items, although this was only a weak trend (F(1,12)  = 2.19, p = 0.16). The left M170 showed no other effects of the stimulus manipulation. In the right hemisphere, there were no M170 effects either in latency or amplitude.

In sum, consistent with the hypothesis that abstract orthographic codes are sound-based, we observed an early effect of form frequency at the visual M100, whether or not the stimuli were presented in their typical script. Thus at the M100, script typicality had no impact on processing, suggesting that at this processing stage, script-invariant form representations are active. Lexical decision times to atypically presented words were, however, significantly delayed, showing that at later stages of processing, atypical presentation does create some type of interference. As discussed above, a previous ERP study did not find evidence for such interference at the N170 stage when kanji words were presented in katakana [20]. However, in our data, M170 latencies did show a slight delay for atypically presented items, raising the possibility that the representations active at the M170 may not be script-invariant, even if the ones encoded at the M100 are. One possibility is that the M100 generator encodes abstract sound-based character/letter string representations while the M170 generator accesses script specific visual word forms. The difference between our M170 and the N170 findings in [20] could be due to many factors, as the two studies used both different brain measures and different types of analyses. For example, one might speculate that the better spatial accuracy of MEG allowed the detection of the small left lateral latency difference, whereas in EEG, the left and right generators of the N170 topography are likely to blend together more, making it harder to detect small monolateral effects.

## Discussion

This research aimed to assess the role of sound in abstract orthographic representations. Is a letter ultimately a visual object, tied to a specific orthography/script, or a mapping between a sound and all its possible visual encodings? Our masked priming and MEG results suggest that sound-identity is what matters: the form representation of a word can be activated just as efficiently whether it is presented in its usual orthographic form or in a different script, in a form it never appears in.

One important question in interpreting our results pertains to the visual relationship between the katakana and hiragana syllabaries. Although the vast majority of katakana and hiragana characters are visually quite distinct (see the appendices for the full stimulus materials), there are a few syllables whose katakana and hiragana are somewhat similar, for example/u/(‘
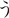
’ in hiragana, ‘
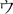
’ in katakana) and/ka/(‘
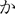
’ in hiragana, ‘
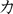
’ in katakana). Importantly though, it is highly unlikely that the hiragana and katakana versions of any particular word would share more than a few similar strokes. There are no reports of this type of visual similarity eliciting masked priming effects, and even if such effects were possible, it would be highly unlikely that they would be equal in size to the effect of full repetition, as we found in Experiment 1a. Further, it would be extremely difficult to explain our MEG results in terms of this subtle similarity. Such an account would have to stipulate that the M100 frequency effect obtained for the atypical items is driven by the subset of the items in which stroke similarity occurs, and that for those items, this subtle similarity is powerful enough to activate the unseen typical word form to such an extent that the amplitude of the M100 response reflects the frequency of the unseen form. While this remains a logical possibility, it is not consistent with the form priming literature at large, in which even substantial form overlap often does not result in masked priming effects [5], [26], [27].

As regards the sound-based hypothesis of orthographic representation, an important goal for future research will be to delineate its visual and phonological limits. For example, there is already evidence that size-invariant ERP effects of masked priming start at 150 ms after target onset when the prime and target are both in the common Arial front, but 100 ms later when the prime is in Arial and the target in the unusual Gigi font [28]. A strong version of the sound-based hypothesis would predict no such discrepancy. However, there is an important difference between this ERP study and the current research, in that there is nothing unusual about the visual appearance of hiragana characters for Japanese speakers, whereas the curly style Gigi-font is relatively unfamiliar to readers of English. The only unfamiliar aspect of our stimuli was that the specific words had no visual frequency in hiragana. The ERP results of [28] were argued to indicate that shape invariance is achieved later than size invariance. An alternative suggested by the present findings is that shape invariance is achieved early, at the M100 level, but the representations encoded at this stage are mappings between sounds and those symbols that are conventionally associated with each sound, including representations of the sound in different scripts as well as upper and lower case versions. Departing substantially from the conventional appearance of a symbol might then delay recognition, resulting in later electromagnetic effects.

On the sound side, a strong version of the sound-based hypothesis would predict that in a language like English, where the orthography-to-phonology mapping is non-transparent, abstract letter representations should be substantially more complicated than in a language such as Finnish, where sound-to-symbol correspondences are almost completely one-to-one. For example, in English, ‘s’ would be a letter, ‘h’ would be a letter, but ‘sh’ would also be a “letter,” since this sequence expresses the single sound/∫/. Further, English would have several symbols that simultaneously participate in multiple abstract letters representations; for example, ‘c’ would be part of the representation that maps the/k/sound to symbols (cat) as well as the representation that maps the/s/sound to symbols (ceiling). The empirical predictions of this hypothesis remain to be tested.

Regarding the time-course of visual word form activation, we found a script independent effect of lexical frequency already at the visual M100. Given the evidence that the M170 is the first component encoding visual word forms [17], [18], the most likely interpretation of our M100 frequency effect is, in fact, not in terms of lexical frequency, but rather in terms of slightly smaller chunks, such as bigrams or trigrams. This can be understood in terms of the model proposed by Dehaene et al. [29], in which the fusiform visual word-form region is not functionally homogenous, but rather exhibits a gradient of increased sensitivity to larger and higher-level components as one moves from more posterior structures to more anterior ones. Thus our data can be taken as evidence for script invariance at the lower levels of this gradient. Interestingly, at the M170 level, we did find an effect of script typicality, suggesting that slightly higher level representations might not be script invariant. This, however, does not rule out the possibility that representations at the M170 level could still be case invariant, as suggested by the fMRI literature on the M170 generating region [30]. Under this hypothesis, we might also have expected a frequency effect at the M170 for the typically presented stimuli, which was, however, not obtained. Although effects of whole-word-form frequency at the M170 level have been reported in some studies [31], [32], most studies have failed to obtain such effects [e.g., 18,33,34,35]. Thus the nature of frequency effects at this level remains somewhat unclear.

In sum, our research aimed to assess the role of sound in orthographic representations. Taking advantage of the multiple scripts of Japanese, we tested whether presenting a word in a script it never appears in would activate the visual word form of that word just as efficiently as showing the word in its usual form. A combination of masked priming and MEG results confirmed this to be the case, suggesting that orthographic representations are fundamentally sound-based. If a strong version of this hypothesis is true, it would have important consequences for theories of letter/character representation, since under this account, letters as mental objects would be primarily phonological representations. In some sense, they would be properties of phonological forms, encoding how a sound representation can be expressed visually.

## Supporting Information

Appendix S1 Stimuli of Experiments 1a and 1b.
(0.09 MB PDF)

Appendix S2 Stimuli of Experiment 2.
(0.07 MB PDF)
