# Supplementary material for: The Nature of Abstract Orthographic Codes: Evidence from Masked Priming and Magnetoencephalography
Source: PLoS One. 2010 May 25;5(5):e10793. doi: 10.1371/journal.pone.0010793 (PMC2876033; doi:10.1371/journal.pone.0010793)
Supplement: Appendix S1 — Stimuli of Experiments 1a and 1b. (0.09 MB PDF) [file pone.0010793.s001.pdf]

Appendix 1. Stimuli of Experiments 1a and 1b.

**Target**	Pronunciation	Translation	**Script**	**Semantic**	Pronunciation	Translation	**Unrelated**	Pronunciation	Translation
カシス	kashisu	currant	かしす	ザクロ	zakuro	pomegranate	メイク	meiku	make up
カナッペ	kanappe	canapé	かなっぺ	オードブル	oodoburu	hors devours	タクシー	takushii	taxi
ブルマ	buruma	shorts	ぶるま	パンツ	pantu	pants	コンビニ	konbini	convenience store
モヘア	mohea	fir	もへあ	カシミア	kashimia	cashmere	トイレ	toire	toilet
スポイト	supoito	dropper	すぽいと	ビーカー	biikaa	beaker	バス	basu	bus
マロン	maron	chestnut	まろん	ヤシ	yashi	palm	エアコン	eakon	air conditioner
スパナ	supana	screw wrench	すぱな	レンチ	renchi	wrench	ベッド	beddo	bed
ラムネ	ramune	lemon soda	なむね	ソーダ	sooda	soda	ビデオ	bideo	video
ズック	zukku	rubber shoes	ずっく	シューズ	shuuzu	shoes	ラジカセ	rajikase	radio
カルキ	karuki	chlorine	かるき	ホルマリン	horumarin	formaldehyde	ネクタイ	nekutai	neck tie
ペンチ	penchi	pliers	ぺんち	ドリル	doriru	drill	ベルト	beruto	belt
クロール	kurooru	stroke	くろーる	バタフライ	batafurai	butterfly	ジャージ	jyaaji	workout pants
バリカン	barikan	hair clipper	ばりかん	ドライヤー	doraiaa	dryer	ブラウス	burausu	blouse
チョッキ	chokki	vest	ちょっき	ジャケット	jyaketto	jacket	カメラ	kamera	camera
グラタン	guratan	gratin	ぐらたん	ドリア	doria	doria	タバコ	tabako	tobacco
ビンタ	binta	slap	びんた	パンチ	panchi	punch	シャンプー	shanpuu	shampoo
ラッコ	rakko	sea otter	らっこ	あざらし	azarashi	sea lion	スプーン	supuun	spoon
カステラ	kasutera	pound cake	かすてら	タルト	taruto	tart	ストロー	sutoroo	straw
ピエロ	piero	clown	ぴえろ	タレント	tarento	performer	フォーク	fooku	fork
トナカイ	tonakai	reindeer	となかい	カモシカ	kamoshika	antelope	ビール	biiru	beer
ゼッケン	zekken	singlet	ぜっけん	レッテル	retteru	label	サザエ	sazae	turban shell
テロップ	teroppu	subtitle	てろっぷ	タイトル	taitoru	title	キンピラ	kinpira	fry
ブランコ	buranko	swing	ぶらんこ	シーソー	shiishoo	see saw	ステーキ	suteeki	steak
メルヘン	meruhen	fairy tale	めるへん	ロマン	roman	romantic	シチュー	shichuu	stew
ブリキ	buriki	tin	ぶりき	ブロンズ	buronzu	bronze	ピザ	piza	pizza
コンロ	konro	stove	こんろ	オーブン	oobun	oven	コイン	koin	coin
カイワレ	kaiware	radish sprouts	かいわれ	アブラナ	aburana	oilseed rape	プレゼント	purezento	present
スコップ	sukoppu	hand shovel	すこっぷ	ショベル	shoveru	shovel	キャベツ	kyabetsu	cabbage
ゴキブリ	gokiburi	cockroach	ごきぶり	セミ	semi	cicada	ニュース	nyuusu	news
ボンベ	bonbe	cylinder	ぼんべ	タンク	tanku	tank	テアトル	teatoru	theater
ミイラ	miira	mummy	みいら	ドクロ	dokuro	scull	セレブ	serebu	celebrity
デマ	dema	rumor	でま	ゴシップ	goshippu	gossip	ピクニック	pikunikku	picnic
アジト	ajito	hideout	あじと	ベース	beesu	base	クレープ	kureepu	crepe
デッサン	dessan	sketch	でっさん	イラスト	irasuto	illustration	ペット	petto	pet
ゲレンデ	gerende	slope	げれんで	アリーナ	ariina	arena	シェフ	shefu	chief
オオカミ	ookami	Japanese wolf	おおかみ	ウルフ	urufu	wolf	ハンコ	hanko	stamp
ピーマン	piiman	green pepper	ぴーまん	トマト	tomato	tomato	エンジン	enjin	engine
ミシン	mishin	sewing machine	みしん	アイロン	airon	iron	カヤック	kayakku	kayak
アトリエ	atorie	atelier	あとりえ	スタジオ	sutajio	studio	バトン	baton	baton
カルテ	karute	medical chart	かるて	ファイル	fairu	file	トリビア	toribia	trivia
バッジ	bajji	button	ばっじ	ブローチ	buroochi	brooch	アリバイ	aribai	alibi
ズボン	zubon	pants	ずぼん	キュロット	kyurotto	curettes	ガム	gamu	gum
パン	pan	bread	ぱん	ライス	raisu	rice	ジャンル	jyannru	genre
パチンコ	pachinko	slot machine	ぱちんこ	バクチ	bakuchi	gamble	トンネル	tonnneru	tunnel
ガット	gatto	gut (tennis)	がっと	ストリング	surorinngu	string	ハンドル	handoru	handle
