# Supplementary material for: The Nature of Abstract Orthographic Codes: Evidence from Masked Priming and Magnetoencephalography
Source: PLoS One. 2010 May 25;5(5):e10793. doi: 10.1371/journal.pone.0010793 (PMC2876033; doi:10.1371/journal.pone.0010793)
Supplement: Appendix S2 — Stimuli of Experiment 2. (0.07 MB PDF) [file pone.0010793.s002.pdf]

Appendix 2. Stimuli of Experiment 2.

Katakana (Typical)	Hiragana (Atypical)	Pronunciation	Translation
トランプ	とらんぷ	toranpu	playing cards
ミイラ	みいら	miira	mummy
ゴンドラ	ごんどら	dondora	gondola
デマ	でま	dema	rumor
アジト	あじと	ajito	hideout
デッサン	でっさん	dessan	sketch
ゲレンデ	げれんで	gerende	slope
ランドセル	らんどせる	randoseru	school bag
オオカミ	おおかみ	ookami	Japanese wolf
ピーマン	ぴーまん	piiman	green pepper
レッテル	れってる	retteru	label
ミシン	みしん	mishin	sewing machine
アトリエ	あとりえ	atorie	atelier
プレハブ	ぷれはぶ	purehabu	house
コンセント	こんせんと	konsento	plug
カルテ	かるて	karute	medical chart
バッジ	ばっじ	bajji	button
シール	しーる	shiiru	sticker
ナトリウム	なとりうむ	natoriumu	sodium
アイヌ	あいぬ	ainu	Ainu
ズボン	ずぼん	zubon	pants
ワクチン	わくちん	wakuchin	vaccine
ウラン	うらん	uran	uranium
オウム	おうむ	oumu	owl
パン	ぱん	pan	bread
コンクール	こんくーる	konkuuru	contest
パチンコ	ぱちんこ	pachinko	slot machine
アルバイト	あるばいと	arubaito	side job
ガット	がっと	gatto	gut (tennis)
アンケート	あんけーと	ankeeto	survey

Katakana (Typical)	Hiragana (Atypical)	Pronunciation	Translation
シミーズ	しみーず	shimiizu	under slips
プロマイド	ぷろまいど	puromaido	profile picture
カシス	かしす	kashisu	currant
カナッペ	かなっぺ	kanappe	canapé
ホッチキス	ほっちきす	hochikisu	staple
ビューラー	びゅーらー	byuuraa	curler
マドロス	まどろす	madorosu	sailor
ブルマ	ぶるま	buruma	shorts
モヘア	もへあ	mohea	fir
スポイト	すぽいと	supoito	dropper
マロン	まろん	maron	chestnut
ポンカン	ぽんかん	ponkan	orange
ムニエル	むにえる	munieru	meuniere
ペチカ	ぺちか	pechika	stove
スパナ	すぱな	supana	screw wrench
ラムネ	なむね	ramune	lemon soda
バギー	ばぎー	bagii	stroller
ズック	ずっく	zukku	rubber shoes
カルビ	かるび	karubi	rib
カルキ	かるき	karuki	chlorine
ペンチ	ぺんち	penchi	pliers
ヤニ	やに	yani	tar
クロール	くろーる	kurooru	stroke
バリカン	ばりかん	barikan	hair clipper
チャック	ちゃっく	chakku	zipper
チョッキ	ちょっき	chokki	vest
シューマイ	しゅーまい	shuumai	dumpling
オブラート	おぶらーと	oburaato	candy coat
ポシェット	ぽしぇっと	boshetto	bag
グラタン	ぐらたん	guratan	gratin
